# 马钱子碱通过阻滞细胞周期抑制人肺癌细胞株PC-9增殖

**DOI:** 10.3779/j.issn.1009-3419.2014.06.02

**Published:** 2014-06-20

**Authors:** 苗 李, 平 李, 梅 张, 峰 马, 丽 苏

**Affiliations:** 230001 合肥，安徽医科大学附属省立医院中医肿瘤科 Department of Chinese Medicine Tumor, the Affiliated Provincial Hospital of Anhui Medical University, Hefei 230001, China

**Keywords:** 马钱子碱, 细胞周期, 人肺癌细胞株PC-9, 增殖, Brucine, Cell cycle, Human lung cancer cell line PC-9, Proliferation

## Abstract

**背景与目的:**

已有的研究表明：Cyclin D1和Cyclin E是细胞周期中重要的正性调控因子，其高表达与肿瘤的增殖密切相关。本研究旨在探讨马钱子碱（Brucine）对人肺癌细胞株PC-9增殖的影响，及其与Cyclin D1和Cyclin E表达的影响。

**方法:**

将PC-9细胞分为4组：空白对照组、DMSO对照组（2‰）、150 μM Brucine组、300 μM Brucine组。CellTiter-Glo发光法、平板克隆形成实验观察该药对PC-9细胞增殖的影响，流式细胞仪检测细胞周期，qRT-PCR检测细胞周期相关基因Cyclin D1、Cyclin E mRNA的表达，Western blot检测细胞周期相关基因Cyclin D1、Cyclin E蛋白的表达。

**结果:**

与对照组比较，CellTiter-Glo发光法、平板克隆形成实验结果显示：Brucine可以抑制人肺癌细胞株PC-9的增殖，并呈时间-剂量依赖性（*P* < 0.01）；流式结果显示对细胞周期的影响主要是阻滞PC-9细胞于G_0_/G_1_期；qRT-PCR结果显示Cyclin D1、Cyclin E mRNA的表达下调；Western blot结果显示Brucine使Cyclin D1、Cyclin E的表达降低。

**结论:**

Brucine能明显抑制人肺癌细胞株PC-9的增殖，机制主要与其通过下调Cyclin D1、Cyclin E表达，进而阻滞细胞周期有关。

## Abstract (en)

**Background and objective:**

It has been proven that Cyclin D1 and Cyclin E are the important positive regulators of cell cycle, they are closely related to the tumor proliferation. The aim of this study is to explore the relationship between Brucine and the proliferation in human lung cancer cell line PC-9, and the effect of it on the expression of Cyclin D1 and Cyclin E.

**Methods:**

PC-9 cells were divided to 4 groups: the normal control group, the DMSO control group (2‰), the 150 μM Brucine group, and the 300 μM Brucine group. The proliferation rate of PC-9 cells was determined by The CellTiter-Glo Luminescent Cell Viability Assay and Colony Formation assay. The change of cell cycle was detected by Flow cytome try. Expressions of cell cycle regulators Cyclin D1, Cyclin E mRNA were determined by qRT-PCR. Protein expression of cell cycle regulators Cyclin D1, Cyclin E were determined by Western blot.

**Results:**

Compared with the control, Brucine remarkably inhibited the proliferation of PC-9 cells in a dose- and time-dependent manner (*P* < 0.01); Flow cytome try showed that Brucine blocked the cell cycle of PC-9 cells at G_0_/G_1_, and the differences were statistically significant (*P* < 0.01); qRT-PCR showed that the expression of Cyclin D1, Cyclin E mRNA were down-regulated; Western blot showed that the protein expression of Cyclin D1, Cyclin E were down-regulated.

**Conclusion:**

Brucine can inhibit the proliferation of human lung cancer cell line PC-9 mainly by blocking the cell cycle at G_0_/G_1_ via down-regulating the expression of Cyclin D1, Cyclin E.

肺癌是影响我国最常见的恶性肿瘤之一，且发病率和死亡率呈逐年上升趋势^[1]^，尽管目前治疗肺癌的方法较多，尤其目前靶向治疗对于表皮生长因子受体（epidermal growth factor receptor, *EGFR*）突变型肺癌有很好疗效，但是由于患者对各种治疗表现出的耐药性及部分*EGFR*突变患者无法接受价格昂贵的靶向制剂导致治疗失败，5年生存率仍然较低^[2]^，其至今仍为人类最难治疗的恶性肿瘤之一^[3]^，因此寻求新的药物治疗肺癌从而改善患者预后显得尤为迫切^[4]^。

祖国医学博大精深，马钱子为马钱科植物马钱或皮氏马钱的成熟种子，为辛温大毒之品，广泛应用于各种肿瘤的临床治疗。其主要有效成份为士的宁（Strychnine）、马钱子碱（Brucine）等吲哚型生物碱^[5]^。近年来诸多研究^[6-16]^提出中药单体马钱子碱无论是体内试验还是体外实验，都表现出对多种肿瘤细胞有明显的抗肿瘤作用，并且涉及到多种抗肿瘤作用机制，但是有关马钱子碱对于*EGFR*突变型肺癌细胞PC-9的作用及其可能的机制，目前均未见报道。因此本研究以*EGFR*突变型肺癌细胞PC-9为研究对象，观察马钱子碱对PC-9细胞的体外作用。

## 1 材料与方法

### 1.1 材料

Brucine购自美国Sigma，用DMSO溶解Brucine配成150 mM，-20 ℃贮存备用（避光）。人肺癌细胞株PC-9由北京生命科学研究所陈良实验室惠赠。RPMI-1640培养液和小牛血清购自美国GIBCO公司；Annexin V-FITC/PI细胞凋亡检测试剂盒购自美国Sigma公司；CellTiter-Glo购自美国Promega公司；Trizol总RNA提取试剂购自Invitrogen公司；逆转录试剂盒、PCR试剂盒购自大连宝生物（TaKaRa）公司；RT^2^ SYBR^®^ Green qPCR Mastermix购自美国SABiosciences公司；Rabbit anti-Cyclin D1、Rabbit anti-Cyclin E购自美国Cell Signaling Technology公司；二抗购自美国Sigma公司。

### 1.2 方法

#### 1.2.1 CellTiter-Glo发光法（The CellTiter-Glo Luminescent Cell Viability Assay）检测细胞活力

取对数生长期细胞，轻轻吹打制成5×10^4^/mL的单细胞悬液，每孔100 μL接种于96孔板中，细胞贴壁后弃上清。各组分别加入100 μL含Brucine 800 μM、400 μM、200 μM、100 μM、0 μM的1640培养液，每组5个平行孔，同时设不含药物的正常对照孔（对照），等体积的DMSO溶剂对照孔（DMSO）和空白对照孔。分别继续培养24 h、48 h、72 h。实验结束前，每孔加入100 μL CellTiter-Glo（提前24 h 4 ℃解冻），于振荡仪上震荡10 min，用酶标仪在Luminescent模式下检测各孔的吸光度（A），用空白对照组调零，据各孔的吸光度计算各组细胞的增殖抑制率。应用直线回归法计算半数抑制浓度（concentration of 50% inhibition, IC_50_）值。

增殖抑制率（inhibitory rate, IR/%）=（对照组A-实验组A）/对照组A×100%。

#### 1.2.2 细胞平板克隆实验（Colony formation assay）检测细胞克隆形成能力

取对数生长期的细胞，消化后计数，每组平行设三个孔，细胞按500个/孔接种于6孔板中。24 h后加入含Brucine 150、300 μM的1640培养基，同时设空白对照组和DMSO对照组，药物干预48 h后，更换为无药物的培养基，继续培养7 d，用2%结晶紫染色，并拍照统计。

#### 1.2.3 PI染色检测细胞周期

收集对照组及处理组细胞，用预冷的PBS洗2次，弃上清，用预冷70%乙醇制备成单细胞悬液，4 ℃固定。24 h后离心，弃上清，PBS漂洗2次，100 μL RNase A 37 ℃水浴30 min；再加入5 μL PI染色混匀，4 ℃避光30 min。用200目尼龙滤膜过滤后进行流式细胞仪检测。实验数据用FlowJo7.6.1进行分析，统计出G_0_/G_1_期的细胞比例。

#### 1.2.4 RNA的提取、反转录、实时荧光定量PCR

使用Trizol试剂盒提取对照和处理组细胞RNA，使用Bio-Rad的iScript cDNA Synthesis Kitd将抽得的RNA进行反转录。取反转产物3 μL作为模板，用SYBR Premix Ex Taq Ⅱ试剂盒（TaKaRa公司）进行qRT-PCR检测，以GAPDH为内参，采用引物如下（5’-3’）（北京生命科学研究所生物制品中心合成）：Cyclin D1(human) Forward：CCGTCCATGCGGAAGAT；Reverse：ATGGCCAGCGGGAAGAC。Cyclin E(human) Forward：ACCAGTTTGCGTATGTG；Reverse：TGTGGGTCTGTATGTTGTG。GAPDH(human) Forward：TGACAACTTTGGTATYCGTGGAAGG；Reverse：AGGCAGGGATGATGTTCTGGAGAG。所有反应采用3复孔，采用7500Fast Real-Time PCR System进行检测，反应条件为预变性95 ℃ 2 min；PCR反应95 ℃ 10 s；60 ℃ 15 s，40个循环。溶解曲线设置：Melt curve：65.0 ℃-95.0 ℃，increment 0.5 ℃ for 0.05 min+plate read。按照2^-ΔΔCt^法计算出各基因相对表达量。

#### 1.2.5 Western blot检测

用裂解液处理收集细胞，以蛋白提取液样品作SDS-PAGE电泳后转膜，封闭后加入一抗（抗体稀释浓度为1:800），4 ℃过夜，TBST洗膜4次，每次10 min；加入二抗（抗体稀释浓度为1:5, 000），TBST洗4次，每次10 min；浸于适量ECL化学发光试剂中（A液:B液=1:1）3 min，取出，室温条件下干燥并用保鲜膜包置于暗盒中，压片曝光3 min-10 min，洗片。

#### 1.2.6 统计学方法

实验数据采用SPSS 19.0软件进行分析，以Mean±SD表示，检验水准α=0.05，以*P* < 0.05为差异有统计学意义。

## 2 结果

### 2.1 Brucine抑制PC-9细胞的生长（表 1）

**1:** 不同浓度Brucine干预PC-9 CellTiter-Glo结果 CellTiter-Glo results of defferent concerntration of Brucine inhibiting PC-9

Group	Brucine concentration (*μ*M)					
	0 (Control)	0 (DMSO)	100	200	400	800
24 h A	0.41±0.01	0.40±0.02	0.36±0.01^*^	0.30±0.02^*^	0.25±0.01^*^	0.15±0.02^*^
IR/%		2	13	26	40	63
48 h A	0.54±0.02	0.53±0.01	0.36±0.03^*^	0.26±0.01^*^	0.16±0.03^*^	0.06±0.01^*^
IR/%		2	33	52	69	89
72 h A	0.82±0.01	0.81±0.02	0.43±0.02^*^	0.28±0.03^*^	0.16±0.02^*^	0.04±0.02^*^
IR/%		1	48	66	80	96
IR: inhibitory rate. ^*^：*P* < 0.05 *vs* control.						

由表 1可知，PC-9细胞被不同浓度的Brucine处理24 h、48 h、72 h后，细胞活力随着作用浓度的升高以及作用时间的延长而明显降低，Brucine对其增殖抑制作用越来越强，且抑制的程度与作用的浓度和时间成正相关。用药组与对照组相比，差异均有统计学意义（*P* < 0.01），但DMSO组与对照组相比*P* > 0.05。用直线回归法测得24 h、48 h、72 h的IC_50_分别为555.3 μM、251.5 μM、136.6 μM。

### 2.2 Brucine对肺癌细胞PC-9集落形成能力的影响（图 1）

**1:**
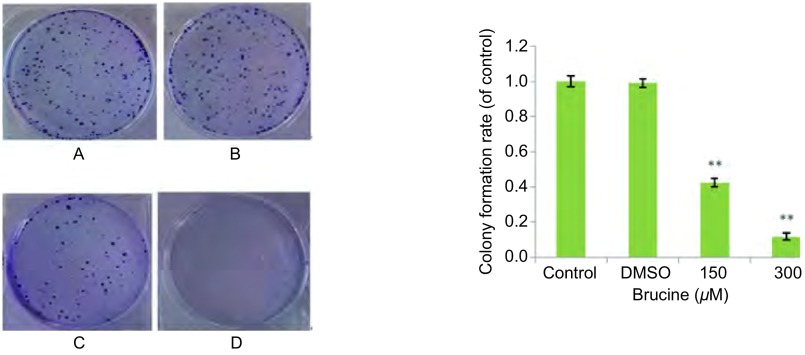
马钱子碱抑制人肺癌细胞株PC-9克隆形成。A：对照；B：DMSO；C：150 *μ*M；D: 300 *μ*M。与对照组比较，^**^：*P* < 0.01。 Brucine inhibited the colony formation of human lung cancer cell line PC-9. A: control; B: DMSO; C: 150 *μ*M; D: 300 *μ*M. Compared with control, ^**^: *P* < 0.01.

各组PC-9细胞分别干预48 h后，更换为无药物的培养基，继续培养7 d，用2%结晶紫染色，于显微镜下观察直径≥200 μm的克隆数，结果显示：对照组、DMSO组、150 μM Brucine组、300 μM Brucine组相对克隆形成率分别为：1.00±0.03、0.99±0.02、0.42±0.02、0.12±0.02。用药组与对照组相比，差异均有统计学意义（*P* < 0.01)，但DMSO组与对照组相比*P* > 0.05。

### 2.3 Brucine对肺癌细胞PC-9周期的影响（图 2）

**2:**
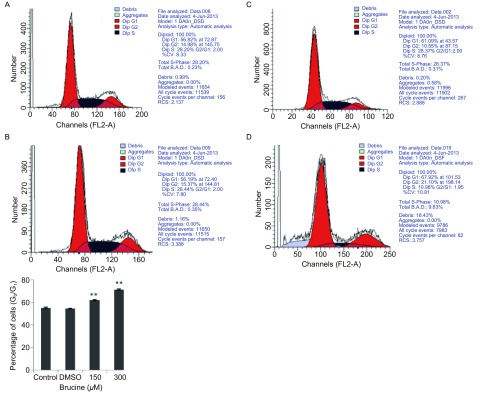
马钱子碱阻滞人肺癌细胞株PC-9于G_0_/G_1_期。A：对照；B：DMSO；C：150 *μ*M；D：300 *μ*M。与对照组比较，^**^：*P* < 0.01。 Brucine blocked the cell cycle at G_0_/G_1_ in human lung cancer cell line PC-9. A: control; B: DMSO; C: 150 *μ*M; D: 300 *μ*M. Compared with control, ^**^: *P* < 0.01.

对照组、DMSO组、150 μM Brucine组、300 μM Brucine组处于细胞周期G_0_/G_1_所占细胞比例分别为：56.82±0.59、56.02±0.50、61.09±0.25、70.31±0.55。用药组与对照组相比，差异均有统计学意义（*P* < 0.01)，但DMSO组与对照组相比*P* > 0.05。结果表明Brucine能将增殖过程中的PC-9细胞阻滞于G_0_/G_1_期。

### 2.4 Brucine对PC-9细胞Cyclin D1、Cyclin E mRNA表达的影响（图 3）

**3:**
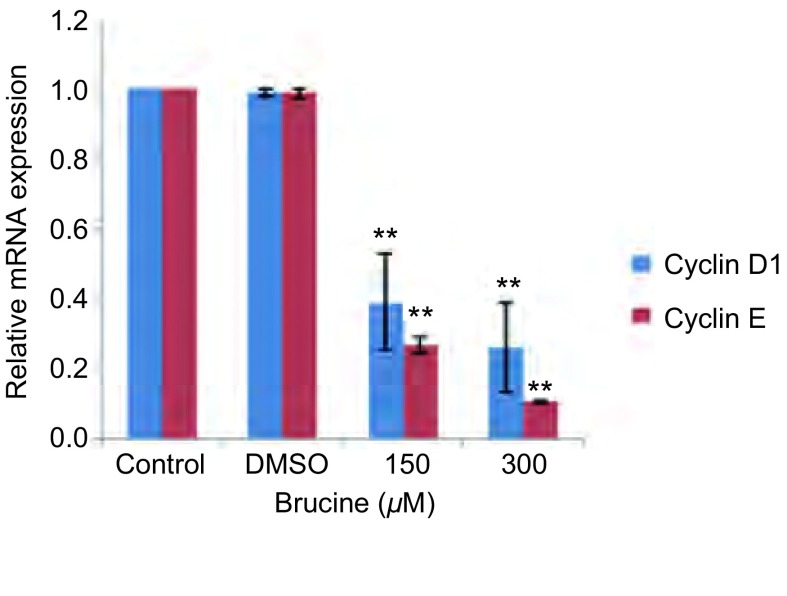
马钱子碱下调肺癌细胞株PC-9周期相关基因Cyclin D1、Cyclin E mRNA表达。与对照组比较，^**^：*P* < 0.01。 Expression of cell cycle regulators Cyclin D1, Cyclin E mRNA were down-regulated in human lung cancer cell line PC-9 by Brucine. Compared with control, ^**^: *P* < 0.01.

qRT-PCR检测结果显示：与对照组相比，DMSO组、150 μM Brucine组、300 μM Brucine组中Cyclin D1、Cyclin E的相对表达分别为：0.99±0.01、0.99±0.01；0.39±0.14、0.27±0.02；0.26±0.13、0.11±0.00。除DMSO组与对照组相比*P* > 0.05之外，其余用药组与对照组相比，差异均有统计学意义（*P* < 0.01）。以上结果表明Brucine能明显地下调细胞周期相关基因Cyclin D1、Cyclin E mRNA的表达，且随着药物浓度增高，其下调基因的程度也增加。

### 2.5 Western blot检测结果（图 4）

**4:**
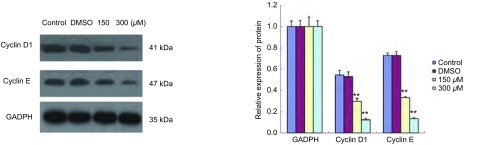
Brucine下调肺癌细胞株PC-9周期相关基因Cyclin D1、Cyclin E蛋白表达.与对照组比较，^**^：*P* < 0.01。 Protein expressions of cell cycle regulators Cyclin D1, Cyclin E were down-regulated in human lung cancer cell line PC-9 by Brucine. Compared with control, ^**^: *P* < 0.01.

分别收集各对照和处理组细胞，提取总蛋白，检测周期相关基因Cyclin D1、Cyclin E蛋白的表达。结果显示，随着Brucine浓度的增高，细胞周期相关基因Cyclin D1、Cyclin E蛋白表达水平逐渐降低。

## 3 讨论

最近研究证实，肿瘤的发生和发展是一个多因素影响的复杂过程^[17]^，涉及多种基因和蛋白的表达异常，但其最终表现均为细胞周期调控机制紊乱、细胞分化受阻^[18]^。增殖是指细胞通过分裂而生成与自身相同的细胞群体的过程。肿瘤细胞的增殖周期与正常细胞一样，要经过G_0_/G_1_期、S期、G_2_/M期等3个时期才能完成一次细胞的增殖过程，任何能阻断这4期中的一期或多期的药物都能有效地抑制肿瘤细胞的增殖生长^[19]^。目前已发现的细胞周期调控因子主要有细胞周期蛋白（Cyclin）、细胞周期蛋白依赖性激酶（cyclin dependent kinase, CDK）、细胞周期蛋白依赖性激酶抑制剂（cyclin-dependent kinase inhibitors, CKI），其中CDK是该调控网络的核心，Cyclin对CDK有正调控作用，在Cyclin系列中，以Cyclin D1研究最多最深入。其为细胞周期中G_1_期进入S期的一个首要调控因子，通过激活CDK4或CDK6等作用，促进DNA合成，加速细胞增殖^[20]^。而在细胞周期中，直接控制细胞周期是否由G_1_期进入S期的重要因子为Cyclin E，研究^[21, 22]^发现，向细胞内注射Cyclin E的抗体能使细胞停滞于G_1_期，说明细胞进入S期需要Cyclin E的参与。其与CDK2结合，促进细胞通过G_1_/S限制点而进入S期。如果Cyclin E合成增加或降解减少，或者Cyclin D1过度表达使细胞周期G_1_/S转换时间缩短，将导致细胞周期加快，肿瘤恶性增生。因此，设法阻滞肿瘤细胞的周期进程已成为一种新的治疗方向。

目前已发现许多通过阻滞细胞周期起到抗肿瘤作用的药物，但是这些药物往往因为副作用较多及癌症患者对其迅速产生的耐药性而限制了其在临床上的运用，尤其对于*EGFR*突变型肺癌，其已对多种化疗药物产生耐药，即使靶向治疗踏上了治疗*EGFR*突变型肺癌的舞台，但是终究因为耐药、价格等各种原因无法普及到每一位*EGFR*突变型肺癌，因此寻找一种抗*EGFR*突变型肺癌细胞且易于被患者接受的药物已成为目前研究的热点。中药单体Brucine因为其广泛抗肿瘤作用而被人熟知，体内实验^[23]^发现Brucine可明显抑制小鼠肉瘤、黑色素瘤、肺癌等的生长；体外实验也发现白血病细胞K562和胃癌C85、HL60、肝癌SMMC-7721、Hep-G2可被其直接抑制；另外现代医学认为肿瘤的发生与人体的免疫监视作用失败有关，诸多化疗药物耐药性的产生也与其损伤机体免疫系统有密切关系，而曲雷鸣等^[24]^发现Brucine不仅不会对免疫系统产生不良影响，而且在与其他化疗药物联合抗癌时反而具有增强免疫功能的作用。即使Brucine的抗肿瘤作用广泛，但对于*EGFR*突变型肺癌细胞的研究仍然是一个未知领域，因此本研究以*EGFR*突变型肺癌细胞PC-9为靶细胞，研究其对PC-9细胞增殖的影响。首先通过CellTiter-Glo发光法和平板克隆形成实验观察了Brucine对PC-9细胞的增殖抑制作用，结果表明Brucine可以浓度和时间依赖性地抑制PC-9细胞的增殖。其次为了探讨Brucine对于PC-9细胞增殖抑制作用的机制，我们通过PI染核流式细胞术分析细胞周期。结果发现：与对照组相比，Brucine可以浓度依赖性的把PC-9细胞阻滞在G_1_期，使之不进入或延迟进入S期，干扰了DNA的合成，并且在300 µM Brucine作用下发现有明显的凋亡峰，这表明Brucine在导致细胞周期G_1_期阻滞时伴随有细胞凋亡，加速抑制了细胞的增殖。为进一步研究Brucine阻滞PC-9细胞周期进程的分子机制，qRT-PCR、Western blot法检测了Brucine对PC-9细胞周期相关基因*Cyclin D1*、*Cyclin E*转录及翻译水平表达的影响，结果发现，随着Brucine浓度的提高，Cyclin D1、Cyclin E mRNA及蛋白表达水平降低。这表明Brucine可能是通过下调Cyclin D1、Cyclin E在EGFR突变型肺癌PC-9细胞中转录及翻译水平的表达来阻滞PC-9细胞于G_0_/G_1_期，这也同时从基因表达的角度反证了Cyclin D1、Cyclin E促进细胞周期G_1_到S期的进程。综上所述，本研究成果为Brucine成为治疗*EGFR*突变型肺癌的新型药物提供理论基础。
